# Utility of laboratory and immune biomarkers in predicting disease progression and mortality among patients with moderate to severe COVID-19 disease at a Philippine tertiary hospital

**DOI:** 10.3389/fimmu.2023.1123497

**Published:** 2023-02-28

**Authors:** Felix Eduardo R. Punzalan, Jaime Alfonso M. Aherrera, Sheriah Laine M. de Paz-Silava, Alric V. Mondragon, Anna Flor G. Malundo, Joanne Jennifer E. Tan, Ourlad Alzeus G. Tantengco, Elgin Paul B. Quebral, Mary Nadine Alessandra R. Uy, Ryan C. V. Lintao, Jared Gabriel L. Dela Rosa, Maria Elizabeth P. Mercado, Krisha Camille Avenilla, Jonnel B. Poblete, Albert B. Albay, Aileen S. David-Wang, Marissa M. Alejandria

**Affiliations:** ^1^ Department of Medicine, Philippine General Hospital, University of the Philippines Manila, Manila, Philippines; ^2^ College of Medicine, University of the Philippines Manila, Manila, Philippines; ^3^ College of Public Health, University of the Philippines Manila, Manila, Philippines; ^4^ Department of Physiology, College of Medicine, University of the Philippines Manila, Manila, Philippines; ^5^ Department of Biology, College of Science, De La Salle University, Manila, Philippines; ^6^ Department of Clinical Epidemiology, Faculty of Medicine and Surgery, University of Santo Tomas, Manila, Philippines; ^7^ Institute of Clinical Epidemiology, National Institutes of Health, University of the Philippines Manila, Manila, Philippines

**Keywords:** biomarkers, cytokines, disease progression, mortality, COVID-19, Filipino

## Abstract

**Purpose:**

This study was performed to determine the clinical biomarkers and cytokines that may be associated with disease progression and in-hospital mortality in a cohort of hospitalized patients with RT-PCR confirmed moderate to severe COVID-19 infection from October 2020 to September 2021, during the first wave of COVID-19 pandemic before the advent of vaccination.

**Patients and methods:**

Clinical profile was obtained from the medical records. Laboratory parameters (complete blood count [CBC], albumin, LDH, CRP, ferritin, D-dimer, and procalcitonin) and serum concentrations of cytokines (IL-1β, IL-2, IL-4, IL-6, IL-8, IL-10, IL-18, IFN-γ, IP-10, TNF-α) were measured on Days 0-3, 4-10, 11-14 and beyond Day 14 from the onset of illness. Regression analysis was done to determine the association of the clinical laboratory biomarkers and cytokines with the primary outcomes of disease progression and mortality. ROC curves were generated to determine the predictive performance of the cytokines.

**Results:**

We included 400 hospitalized patients with COVID-19 infection, 69% had severe to critical COVID-19 on admission. Disease progression occurred in 139 (35%) patients, while 18% of the total cohort died (73 out of 400). High D-dimer >1 µg/mL (RR 3.5 95%CI 1.83–6.69), elevated LDH >359.5 U/L (RR 1.85 95%CI 1.05–3.25), lymphopenia (RR 1.91 95%CI 1.14–3.19), and hypoalbuminemia (RR 2.67, 95%CI 1.05–6.78) were significantly associated with disease progression. High D-dimer (RR 3.95, 95%CI 1.62–9.61) and high LDH (RR 5.43, 95%CI 2.39–12.37) were also significantly associated with increased risk of in-hospital mortality. Nonsurvivors had significantly higher IP-10 levels at 0 to 3, 4 to 10, and 11 to 14 days from illness onset (*p<*0.01), IL-6 levels at 0 to 3 days of illness (*p*=0.03) and IL-18 levels at days 11-14 of illness (*p*<0.001) compared to survivors. IP-10 had the best predictive performance for disease progression at days 0-3 (AUC 0.81, 95%CI: 0.68–0.95), followed by IL-6 at 11-14 days of illness (AUC 0.67, 95%CI: 0.61–0.73). IP-10 predicted mortality at 11-14 days of illness (AUC 0.77, 95%CI: 0.70–0.84), and IL-6 beyond 14 days of illness (AUC 0.75, 95%CI: 0.68–0.82).

**Conclusion:**

Elevated D-dimer, elevated LDH, lymphopenia and hypoalbuminemia are prognostic markers of disease progression. High IP-10 and IL-6 within the 14 days of illness herald disease progression. Additionally, elevated D-dimer and LDH, high IP-10, IL-6 and IL-18 were also associated with mortality. Timely utilization of these biomarkers can guide clinical monitoring and management decisions for COVID-19 patients in the Philippines.

## 1 Introduction

Coronavirus disease 2019 (COVID-19) has taken the world by storm prompting the World Health Organization (WHO) to declare it a pandemic last March 11, 2020. COVID-19 primarily presents as fever, cough, fatigue, and dyspnea, but the clinical presentation can vary, from asymptomatic infection to severe, life-threatening symptoms. Most patients infected with the SARS-CoV-2 experience mild symptoms or remain asymptomatic, while 15% have severe, life-threatening diseases (1). Disease progression has often been linked to acute respiratory distress syndrome (ARDS) and cytokine storm. Furthermore, patients with COVID-19 with comorbidities, such as hypertension, cardiovascular disease, diabetes mellitus, chronic obstructive pulmonary disease (COPD), asthma, and immunocompromising conditions such as human immunodeficiency virus (HIV) infection, chronic steroid use, and active malignancy, are more likely to develop a more severe course and progression of the disease (2, 3). Current evidence points to a dysregulated immune response to the virus causing these known syndromes in COVID-19. Laboratory parameters in COVID-19 also differ with disease severity. Recent meta-analyses have described various biomarkers, such as lymphopenia, thrombocytopenia, elevated procalcitonin (PCT), C-reactive protein (CRP), lactate dehydrogenase (LDH), aspartate aminotransferase (AST), alanine aminotransferase (ALT), serum amyloid A (SSA), D-dimer, ferritin, troponin, B-type natriuretic peptides, creatinine and blood urea nitrogen (BUN), and elevated cytokines, including IL-6, TNF-α, IFN-γ, IL-8, and IL-10, to be associated with worse clinical outcomes and mortality in COVID-19 (4–10).

Most deaths from COVID-19 are from severe and critical diseases. Hence, studies have investigated biomarkers that may be predictive of progression to severe disease and adverse outcomes as shown in **Supplementary Table 1**. Data from a large cohort of patients admitted to a tertiary COVID-19 referral center in the Philippines reported the following factors to be predictive of severe disease: increasing age, diabetes mellitus (DM), chronic kidney disease (CKD), chronic obstructive pulmonary disease (COPD), hypertension, coronary artery disease, or metabolic syndrome. Likewise, patients with severe disease had significantly higher LDH, ferritin, D-dimer, and CRP (11). With the massive effect of cytokines in the development of cytokine storms and other complications in COVID-19, its utility as a predictive biomarker has been explored by several studies. If proven useful, these cytokines would be helpful in early recognition and intervention before disease progression becomes uncontrollable. IL-6 has already been predicted as a useful biomarker in managing COVID-19. Elevations in IL-6 have commonly been reported in several studies and are strongly associated in severe and critically ill patients with an increased risk for ICU admission, respiratory failure, and overall poor prognosis (12–19). Moreover, C-reactive protein (CRP) was also included among the strongest predictors for ICU admission or death at 30 days in COVID-19 (20, 21). Interferon-gamma (IFN-γ) has also been increased in severe cases compared to milder cases or even with healthy controls (4, 15, 19). Early robust IFN-γ response is protective against COVID-19 infection. However, a delayed IFN-γ response did not limit viral load and led to increased inflammation and collateral damage (22). Other chemokines such as CCL2 and CXCL2 are likewise reported to be higher in infected cases (14, 22). Several chemokines, such as CXCL1 and CXCL5, have also been elevated in severe COVID-19 patients (23).

More studies are needed to better understand potential biomarkers that can predict cytokine storm-like syndrome associated with COVID-19 in Filipino population (24). Moreover, there is very limited data regarding genetic difference associated with different disease phenotypes among Filipino COVID-19 patients. Identifying biomarkers that are associated with clinical deterioration may help in treatment decisions. In this study, we studied different biomarkers (complete blood count [CBC], albumin, LDH, CRP, ferritin, D-dimer, and procalcitonin) and cytokines (IL-1β, IL-2, IL-4, IL-6, IL-8, IL-10, IL-18, IFN-γ, IP-10, TNF-α) associated with disease progression and mortality among patients with confirmed moderate to severe COVID-19 infection in the University of the Philippines - Philippine General Hospital (UP-PGH).

## 2 Patients and methods

### 2.1 Study design and setting

This was a prospective cohort study of adults with confirmed moderate to severe COVID-19 infection admitted in the University of the Philippines – Philippine General Hospital (UP-PGH) from October 2020 to September 2021. UP-PGH is a tertiary university hospital and a COVID-19 referral center in the National Capital Region (NCR) where cases of COVID-19 in the Philippines are the highest (25). The conduct of the study was approved by the University of the Philippines Manila Research Ethics Board (UPMREB 2020-251-01).

Potential participants were recruited upon admission through referral from attending physicians. Once consent is obtained, blood samples were collected on specific time points (i.e., days 0 – 3, 4 – 10, 11 – 14, and >14 from the onset of COVID-19 symptoms) to measure serum concentrations of selected pro-inflammatory and anti-inflammatory cytokines, namely IL-1β, IL-2, IL-4, IL-6, IL-8, IL-10, IL-18, IFN-γ, IP-10, TNF-α. The Milliplex Cytokine Storm Panel (Merck) that utilized the Luminex xMAP technology as per manufacturer’s instructions was used for cytokine measurement. We also obtained the results of common laboratory tests requested by attending physicians specifically, CBC, albumin, LDH, CRP, ferritin, D-dimer, and procalcitonin. The primary outcomes of interest were: 1) disease progression and 2) in-hospital mortality. We monitored the occurrence of outcomes daily from enrolment until death or hospital discharge.

### 2.2 Participants

Eligible patients included hospitalized adults (18 years and above) with confirmed COVID-19 infection, classified as moderate or severe disease. We excluded pregnant patients, patients with known immunodeficiencies such as those with active malignancies and autoimmunity, and those receiving immunosuppressive medications. Quota sampling was employed.

### 2.3 Definitions

A *confirmed COVID-19 case* was defined as a patient with laboratory-confirmed positive RT-PCR test for SARS-CoV-2. We used the Philippine COVID-19 Living Recommendations for severity classification (26). *Moderate COVID-19* include those with clinical (cough, fever, and tachypnea) and/or radiographic evidence of pneumonia but without difficulty breathing or shortness of breath, respiratory rate <30 breaths/minute, or peripheral oxygen saturation (SpO2) ≥ 94% on room air. It also includes symptomatic patients without pneumonia but with risk factors for progression namely hypertension, cardiovascular disease, diabetes mellitus, chronic obstructive pulmonary disease (COPD), asthma, and immunocompromising conditions such as human immunodeficiency virus (HIV) infection, chronic steroid use, and active malignancy. *Severe COVID-19* includes those with pneumonia and any of the following: signs of respiratory distress, SpO2 <94% at room air, respiratory rate ≥30 breaths/minute, or requiring oxygen supplementation. Patients with impending respiratory failure requiring high flow oxygen or ventilatory support, ARDS, sepsis, or septic shock, deteriorating sensorium, multi-organ failure, and thrombosis already have *critical COVID-19.*

*Disease progression* is present if any of the following develop during the course of hospitalization: 1) ARDS or worsening of ARDS based on the Berlin Criteria (27), 2) need for mechanical ventilation, 3) acute kidney injury defined as an increase in serum creatinine by >0.3 mg/dL within 48 hours or 1.5 x from baseline occurring within the prior seven days or a decrease in urine output of less than 0.5 mL/kg/h for 24 hours, 4) need for acute renal replacement therapy, 5) symptomatic cerebro- or cardiovascular thrombotic events such as stroke documented by CT-scan, acute myocardial infarction with rise in troponin-I values by >20%, acute limb ischemia, or venous thromboembolism documented by imaging, 6) acute myocarditis with findings of new wall motion abnormalities or worsening ejection fraction to <45%, 7) ICU admission, and 8) in-hospital mortality. *In-hospital mortality* is death from any cause during admission.

### 2.4 Sample size

To detect the association of clinically relevant biomarkers with disease progression and mortality with an odds ratio of at least 2.00, the computed sample size was 400 at an alpha of 0.05 and a beta of 0.20. For the baseline estimate of the risk of death, the 16.7% case-fatality rate in UP PGH was used (28). Estimates for disease progression were based on the reported odds ratio from recent studies (5, 29, 30). The study was powered for most of the clinically usable biomarkers in resource-limited settings based on the Philippine Society for Microbiology and Infectious Diseases (PSMID) Rapid Evidence Reviews on COVID-19 (31).

### 2.5 Data collection

Demographic and clinical data extracted from electronic medical records and the results of routine laboratory tests and cytokine analysis were encoded into a standard data collection form in the Research Electronic Data Capture (REDCap), a secure web-based application commonly used to capture data for clinical research and to create databases (32).

### 2.6 Statistical analysis

Shapiro-Wilk test and graphical representation were done using the data on patients’ age. The data were normally distributed. Demographic and clinical characteristics were compared according to the main outcomes of mortality and disease progression using t-test for continuous variables and Fisher’s exact test for dichotomous variables.

To determine the temporal changes in mean clinical laboratory biomarker levels between the outcome groups, the day of illness was grouped into four distinct time points: day 0-3, day 4-10, day 11-14, and day > 14 from the onset of illness. The mean biomarker levels and their confidence intervals were plotted against these four time points using R.

To determine the association between clinical laboratory biomarker levels and outcomes of interest, the following actions were made. First, for clinical applicability, biomarker levels were dichotomized using the following published cut-offs: NLR >3 (15), D-Dimer >1 µg/mL (33), Ferritin >300 µg/mL (33), hs-CRP >5 mg/dL (18), and LDH >359.50 U/L (34). Second, since patients usually get admitted on the 4^th^ to 10^th^ day of illness, we selected this period as reference for the analysis of prediction of disease progression and mortality. A generalized linear model for the binomial family was used to estimate the risk ratio for disease progression and in-hospital mortality using binary forms of the biomarker as the predictor variables. Separate models were created for each biomarker and each outcome of interest. Analysis was adjusted to age using a cutoff of 55 years, presence of CKD, presence of DM, treatment with tocilizumab, and treatment with dexamethasone during hospitalization. Association between laboratory results and outcomes of interest were reported as risk ratios with 95% confidence intervals.

We used a repeated measures mixed model regression analysis to determine the association between cytokine levels and outcomes over four time points. Main effects (time and outcome) with statistically significant results were subjected to *post-hoc* analysis to identify specific comparison pairs. Dot plots were used to graphically represent the trends in cytokine levels across time points and between groups. Statistical significance was set at a *p*-value of <0.05. The analysis was adjusted for age, comorbidities (CKD, DM), and treatment (tocilizumab, dexamethasone). Data were presented using linear prediction graphs. *Post-hoc* significance similarly used Šidák-Holm adjusted p-values. The prognostic utility of the cytokines in predicting disease progression and mortality was evaluated using ROC curves. Statistical analyses were performed in STATA/IC 15.1, GraphPad Prism version 8 for Windows, and R version 3.3.1.

## 3 Results

### 3.1 Patient demographics and profile

We analyzed 400 hospitalized adult patients with moderate to critical COVID 19. The demographic and clinical characteristics of the patients are summarized in **Table 1**. The mean age of the cohort was 56 years, and majority were males (61%). Hypertension (56%), diabetes mellitus (32%), and cardiac disease (12%) were the most common comorbid conditions. On admission, 123 (31%) had nonsevere disease (moderate COVID-19 infection), while 277 (69%) had severe to critical disease. Common presenting symptoms were cough (74%), shortness of breath (73%), fever (58%), generalized weakness (24%), and decreased appetite (23%). In the cohort, 139 (35%) exhibited disease progression. No comorbidities were associated with disease progression in this cohort. Patients with disease progression were generally older (*p =* 0.01) and presented with shortness of breath (*p* = 0.01) and sensorial changes (altered sensorium, delusions, hallucinations, and behavioral changes) (*p* < 0.001). On the other hand, the in-hospital mortality rate of the cohort was 18%. More patients who died were older (*p* = 0.01), had diabetes (*p* = 0.01), had chronic kidney disease (*p* = 0.03), presented with shortness of breath (*p* = 0.02), and sensorial changes (*p* < 0.001). Many also received therapeutic interventions, namely dexamethasone (78%), remdesivir (56%), tocilizumab (52%), anticoagulants, and antithrombotics (88%). The administration of remdesivir (*p =* 0.001), dexamethasone (*p =* 0.01), tocilizumab (*p* = 0.01), and anticoagulants/antithrombotics (*p =* 0.01) was higher among patients who progressed to the severe-critical stage. Meanwhile, more nonsurvivors received dexamethasone (*p =* 0.03) and tocilizumab (*p =* 0.04). Illness severity on admission was associated with both disease progression and in-hospital mortality (*p* < 0.001). More patients who progressed to more severe disease (82%) or died (86%) already had severe-critical COVID-19 on admission.

**Table 1:** Comparison of demographic and clinical characteristics of hospitalized adult patients with confirmed COVID-19 according to outcomes of disease progression and mortality.

Characteristics	Total (*N*=400)	Disease Progression			Mortality		
		Without disease progression (*n*=261)	With diseaseprogression, (*n*=139)	*p*-value	Survivor (*n*=327)	Non- Survivor (*n*=73)	*p-*value
**Age, years; mean (SD)**	56 (15)	55 (15)	59 (14)	0.01	55 (15)	60 (13)	0.01
**Male, n (%)**	243 (61%)	157 (60%)	86 (62%)	0.75	201 (61%)	42 (58%)	0.60
**Length of hospitalization; mean (SD)**	15.65 (9.63)	15.60 (8.03)	15.75 (12.13)	0.89	16.43 (9.19)	12.18 (10.84)	<0.001
**On Mechanical Ventilation at admission; n (%)**	44 (11)	5 (2)	39 (28)	<0.001	18 (6)	26 (36)	< 0.001
COVID-19 Severity Classification on Admission							
** Nonsevere**	123 (31%)	98 (38%)	25 (18%)	<0.001	63 (35%)	10 (14%)	<0.001
** Severe – Critical**	277 (69%)	163 (62%)	114 (82%)		214 (65%)	63 (86%)	
Comorbidities							
** Diabetes Mellitus**	127 (32%)	75 (29%)	52 (37%)	0.09	94 (29%)	33 (45%)	0.01
** Hypertension**	224 (56%)	140 (54%)	84 (60%)	0.21	179 (55%)	45 (62%)	0.30
** Cardiac disease**	46 (12%)	31 (12%)	15 (11%)	0.87	38 (12%)	8 (11%)	1.00
** Liver disease**	3 (1%)	2 (1%)	1 (1%)	1.00	2 (1%)	1 (1%)	0.45
** Chronic kidney disease**	32 (8%)	16 (6%)	16 (11%)	0.08	21 (6%)	11 (15%)	0.03
** COPD**	4 (1%)	3 (1%)	1 (1%)	1.00	3 (1%)	1 (1%)	0.55
** Asthma**	26 (7%)	18 (7%)	8 (6%)	0.83	19 (6%)	7 (10%)	0.29
** Active tuberculosis**	6 (2%)	5 (2%)	1 (1%)	0.67	5 (2%)	1 (1%)	1.00
** HIV/AIDS**	0 (0%)	0 (0%)	0 (0%)	–	0 (0%)	0 (0%)	–
** Malignancy**	3 (1%)	2 (1%)	1 (1%)	1.00	3 (1%)	0 (0%)	1.00
** Neurologic disease**	14 (4%)	8 (3%)	6 (4%)	0.57	9 (3%)	5 (7%)	0.15
Smoking History							
** Non-smoker**	255 (64%)	167 (64%)	88 (63%)	0.37	208 (64%)	47 (64%)	0.36
** Previous smoker**	111 (28%)	68 (26%)	43 (31%)		88 (27%)	23 (32%)	
** Current smoker**	32 (8%)	24 (9%)	8 (6%)		29 (9%)	3 (4%)	
Treatments/Interventions							
** Dexamethasone**	311 (78%)	193 (74%)	118 (85%)	0.01	247 (76%)	64 (88%)	0.03
** Remdesivir**	224 (56%)	131 (50%)	93 (67%)	0.001	177 (54%)	47 (64%)	0.12
** Tocilizumab**	207 (52%)	122 (47%)	85 (61%)	0.01	161 (49%)	46 (63%)	0.04
** Anticoagulation/antthrombotics**	353 (88%)	223 (85%)	130 (94%)	0.02	285 (87%)	68 (93%)	0.23
** *Convalescent Plasma***	14 (3%)	7 (3%)	7 (5%)	0.26	11 (3%)	3 (4%)	0.73

### 3.2 Trends in laboratory parameters

**Figure 1:**
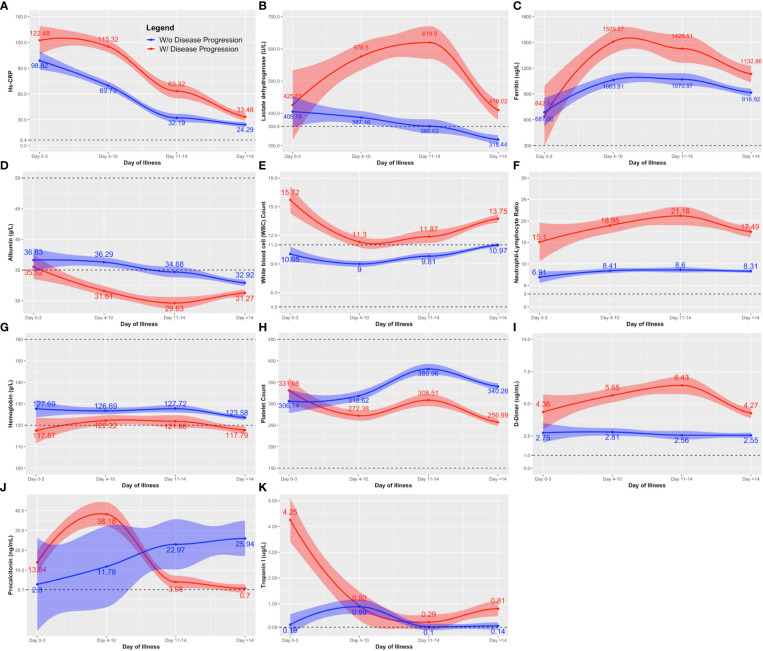
Comparison of the clinical laboratory biomarkers throughout the course of illness between COVID-19 patients with and without disease progression. The clinical biomarkers are: **(A)** hs-CRP; **(B)** lactose dehydrogenase (LDH); **(C)** Ferritin; **(D)** albumin; **(E)** white blood cell (WBC); **(F)** neutrophil-lymphocyte ratio; **(G)** hemoglobin; **(H)** platelet count; **(I)** D-dimer; **(J)** procalcitonin; and **(K)** Troponin. Line estimates were created using Locally Weighted Scatterplot Smoothing (LOWESS) showing 95% CI as shaded area around the line estimate. Points at each day interval represent mean estimate computed at that time point. Broken lines show the normal ranges of the tests.

Serum ferritin, LDH, CRP, and NLR (i.e., inflammatory markers), were elevated throughout the course of illness among those who exhibited disease progression (**Figures 1A–C, F**). Moreover, their WBC counts were consistently above normal (WBC >10^9^/L) compared to those who did not exhibit disease progression (**Figure 1E**). D-dimer, a marker of ongoing activation of the hemostatic system and overall inflammation, also showed a similar pattern as other inflammatory markers (**Figure 1I**). Albumin, a negative acute phase reactant, exhibited a marked decline to below normal levels among those with disease progression (**Figure 1D**). The differences between those who did and did not progress to a more severe disease were more obvious on days 4 to 14 of illness.

Throughout the course of illness, hemoglobin and platelet values were lower among those with disease progression compared to those without (**Figures 1G, H**). Nevertheless, hemoglobin and platelet values remained near normal for both groups (Normal values: Hemoglobin, Male 13.5 – 17.5 g/L, Female 12.0 – 16.0 g/L; Platelet 150 – 399 x 10^9^/L). A distinct peak in the procalcitonin level was observed on days 4-10 of illness, followed by a rapid decline towards the second week among those with disease progression (**Figure 1J**). The opposite was observed among nonprogressors, showing a steady increase in procalcitonin levels. Note that we only had at most 19 procalcitonin test results available for analysis in each observation period. Similarly for troponin, the tests were mostly done on days 4-10, with only 75 test results included in the analysis. Troponin was generally elevated, but the trends did not reveal marked deviation between those who did and did not exhibit disease progression (**Figure 1K**).

**Figure 2:**
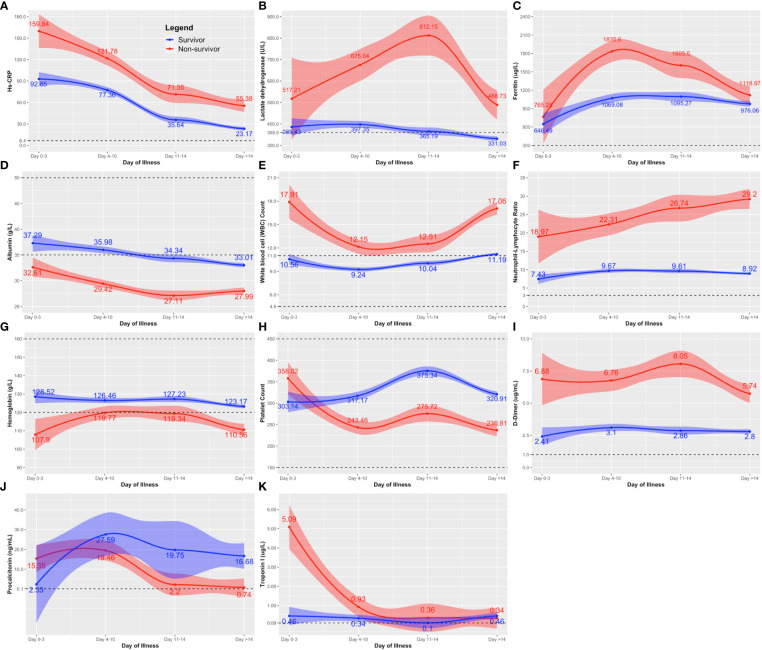
Comparison of the clinical laboratory biomarkers throughout the course of illness between COVID-19 survivors and nonsurvivors. The clinical biomarkers are: **(A)** hs-CRP; **(B)** lactose dehydrogenase (LDH); **(C)** Ferritin; **(D)** albumin; **(E)** white blood cell (WBC); **(F)** neutrophil-lymphocyte ratio; **(G)** hemoglobin; **(H)** platelet count; **(I)** D-dimer; **(J)** procalcitonin; and **(K)** Troponin. Line estimates were created using Locally Weighted Scatterplot Smoothing (LOWESS) showing 95% CI as shaded area around the line estimate. Points at each day interval represent mean estimate computed at that time point. Broken lines show the normal ranges of the tests.

For routine laboratory tests, nonsurvivors exhibited a similar pattern as patients with disease progression (**Figures 1**, **2**), except that anemia was observed among nonsurvivors (**Figure 2G**), and the initial peak in procalcitonin levels was not observed among nonsurvivors (**Figure 2J**).

**Figure 3:**
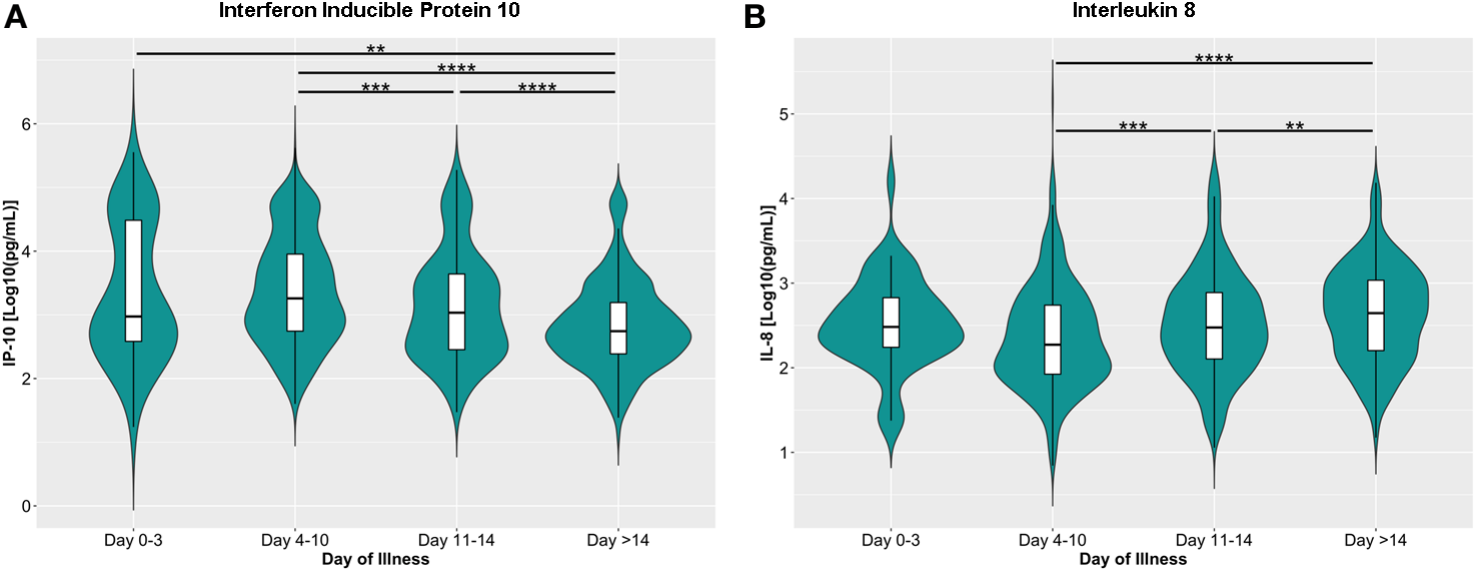
Dynamics of serum levels of **(A)** inducible protein 10 (IP-10) and **(B)** interleukin 8 (IL-8) during the disease course in the COVID-19 patient cohort. The cytokines were measured in terms of days from illness onset. All samples collected from 400 patients were stratified into four intervals starting from illness onset. The dots represent individual measurement, and the box plots represent medians with interquartile range. The different groups were compared using the Kruskal-Wallis test with Dunn’s *post hoc* test. **p<0.01, ***p<0.001, ****p<0.0001.

We also checked the general trend of the serum cytokine levels for all COVID-19 patients included in this study. The serum concentrations of IFN-γ, IL-1β, IL-2, IL-4, IL-6, IL-10, IL-18, and TNF-α did not change significantly throughout the course of illness (**Supplementary Figure 1**). The IP-10, considered to be an “early cytokine”, peaked (1819.70 ng/mL; IQR: 549.54 – 8912.51) within ten days of illness onset and declined steadily thereafter (**Figure 3A**). On the other hand, IL-8, a “late” cytokine, increased after ten days and peaked 14 days after illness onset (446.68 ng/mL; IQR: 158.49 – 1071.52) (**Figure 3B**).

### 3.3 Association of biomarkers with disease progression and mortality

Among the routine laboratory tests taken on days 4 to 10, high NLR (RR 8.49), high D-dimer (RR 4.14), high LDH (RR 2.62), low ALC (RR 2.31), and low albumin levels (RR 3.22) were associated with disease progression (*p* < 0.05). Except for NLR (*p =* 0.10), the associations remained statistically significant after adjustment for age, comorbidities (DM, CKD), and interventions (tocilizumab, dexamethasone). On the other hand, elevated D-dimer, elevated LDH, low albumin, and low ALC were associated with increased risk of in-hospital mortality (*p <*0.05). After adjustment, only high D-dimer and high LDH remained statistically significant. See **Table 2**.

**Table 2:** Association of Selected Biomarkers (taken on Days 4-10) with Disease Progression and In-Hospital Mortality.

Biomarkers	Disease Progression					In-Hospital Mortality				
	*N*	Crude RR (95% CI)	*p*-value	Adjusted RR (95% CI)*	*p*- value	*N*	Crude RR (95% CI)	*p*-value	Adjusted RR (95% CI)*	*p*- value
Absolute Lymphocyte < 1.1×10^3^ cells/μL	233	2.31 (1.39–3.81)	0.001	1.91 (1.14–3.19)	0.01	248	2.39 (1.25–4.56)	0.008	1.85 (0.95–3.59)	0.07
Neutrophil to lymphocyte ratio (NLR) > 3	234	8.49 (1.23–58.48)	0.03	5.21 (0.73–37.13)	0.10	249	5.55 (0.80–38.52)	0.08	3.19 (0.43–23.32)	0.25
D-dimer > 1 µg/mL	206	4.14 (2.16–7.96)	<0.0001	3.50 (1.83–6.69)	<0.0001	219	5.08 (2.07–12.44)	<0.0001	3.95 (1.62–9.61)	0.003
Albumin < 40 g/L	212	3.22 (1.25–8.33)	0.02	2.67 (1.05–6.78)	0.04	227	4.56 (1.15–18.04)	0.03	3.60 (0.91–14.24)	0.07
Lactate dehydrogenase (LDH) > 359.5 U/L	225	2.62 (1.57–4.38)	<0.0001	1.85 (1.05–3.25)	0.03	239	5.22 (2.30–11.85)	<0.0001	5.43 (2.39–12.37)	<0.0001
Serum Ferritin > 574.5 µg/mL	219	1.41 (0.86–2.30)	0.17	0.98 (0.60–1.60)	0.93	233	1.79 (0.91–3.54)	0.10	1.27 (0.63–2.56)	0.50
High-Sensitivity C-Reactive Protein (hsCRP) > 5 mg/dL	188	5.12 (0.76–34.56)	0.09	3.39 (0.47–24.67)	0.23	199	3.62 (0.53–24.75)	0.19	3.27 (0.44–24.55)	0.25

*Adjusted to age using a cutoff of 55 years, presence of CKD, presence of DM, treatment with tocilizumab, and treatment with dexamethasone during hospitalization.

**Figure 4:**
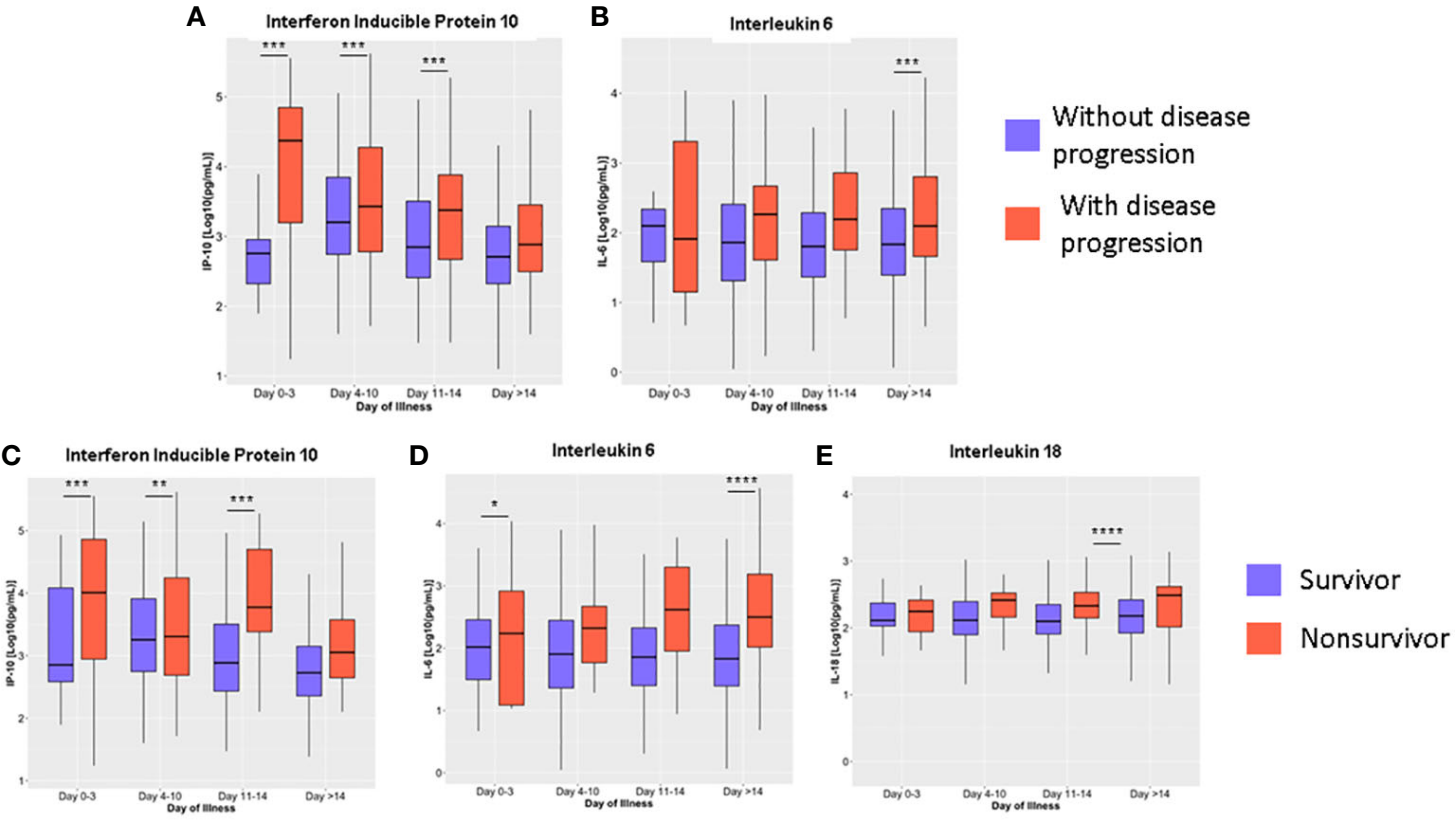
Dynamics of serum cytokine levels during the disease course in COVID-19 patients based on disease progression **(A, B)** and mortality **(C–E)**. The cytokines were measured in terms of days from illness onset. All samples collected from 400 patients were stratified into four intervals starting from illness onset. The dots represent individual measurements, and the box plots represent medians with interquartile range. The different groups were compared by repeated measures mixed model regression with *post hoc* test. *p<0.05, **p<0.01, ***p<0.001, ****p<0.0001.

Repeated measures mixed model regression analysis showed that IP-10 and IL-6 levels were associated with disease progression and mortality, while IL-18 levels were associated only with mortality (**Supplementary Figures 2**-**5**). Generally, the IP-10 levels of COVID-19 patients were significantly higher during the early days of infection and then lowest levels were reached at >14 days from illness onset. IP-10 levels of patients with disease progression were higher at 0 to 3, 4 to 10, and 11 to 14 days from illness onset compared to patients without disease progression (**Figure 4A**). IL-6 levels were higher among those with disease progression (**Figure 4B**) compared to patients without disease progression beyond 14 days of illness onset.

IP-10 levels at 0 to 3, 4 to 10, and 11 to 14 days from illness onset were higher among nonsurvivors compared to survivors (*p <* 0.01) (**Figure 4C**). IL-6 levels of nonsurvivors (2454.46 ng/mL; IQR: 906.30 – 4002.63) were also elevated at 0 to 3 days of illness onset compared to survivors (523.47 ng/mL; IQR: -183.69 – 1230.63) (*p* = 0.03) (**Figure 4D**). For IL-18, levels were significantly higher among nonsurvivors (890.36 ng/mL; IQR: 668.33 – 1112.40) on days 11 to 14 compared to survivors (172.84 ng/mL; IQR: 81.39 – 264.30; *p* < 0.001) (**Figure 4E**). No significant differences were observed for the rest of the cytokines analyzed (**Supplementary Figures 2**, **3**). For both outcomes, we did not observe a change in the statistical significance after adjusting the model for age, comorbidities, and interventions. Adjusted predictions of TNF-α for mortality outcomes could not be evaluated due to non-convergence of data using the adjusted model (**Supplementary Tables 2**, **3**).

### 3.4 Temporal kinetics of biomarkers among COVID-19 patients

No statistical significance was observed when comparing the temporal differences in IL-6 levels among COVID-19 patients without disease progression. However, among COVID-19 patients with disease progression, a significant increase in IL-6 levels from days 4 – 10 (397.65 ng/mL; IQR: 5.55 – 800.86) to days >14 (1248.50 ng/mL; IQR: 798.47 – 1698.52) from illness onset was observed (p < 0.05). For both groups (i.e., with and without disease progression), higher IP-10 levels were observed during the earlier days of illness, days 0 – 3 and 4 – 10, compared to the latter days of illness (days 11 – 14 and days > 14) (**Supplementary Table 2**; **Supplementary Figure 4**).

Among nonsurvivors, significantly higher level of IL-6 was observed at >14 days (3037.35 ng/mL; IQR: 2320.95 – 3753.76) of illness compared to days 4 – 10 (421.17 ng/mL; IQR: -146.30 – 988.63; *p* < 0.001) and 11 – 14 (1014.34 ng/mL; IQR: 389.60 – 1639.08; *p* < 0.001). On the other hand, IL-18 significantly increased and peaked at days 11 – 14 (890.36 ng/mL; IQR: 668.33 – 1112.40) compared to days 4 – 10 (244.08 ng/mL; IQR: 46.92 – 441.25; *p* < 0.001). There was no significant difference in the levels of IL-6 and IL-18 among the survivors. For IP-10, the level peaked at days 0 – 3 (57367.50 ng/mL; IQR: 36903.53 – 77831.46) and was significantly higher compared to days 4 – 10 (24424.94 ng/mL; IQR: 16597.45 – 32252.43; *p* < 0.05) and >14 days of illness (36941.07 ng/mL; IQR: 28421.85 – 45460.29; *p* < 0.001) (**Supplementary Table 3**; **Supplementary Figure 5**).

### 3.5 Predictive performance of cytokines

**Figure 5:**
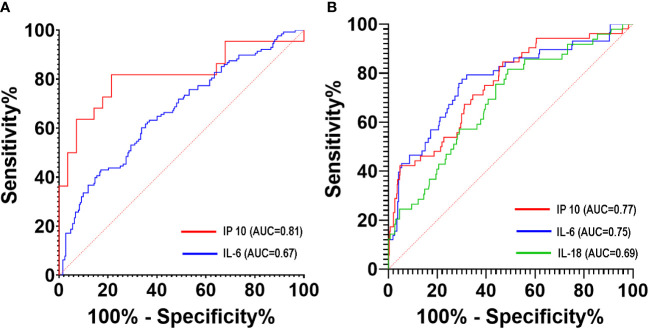
ROC curves of serum cytokine levels to predict disease progression **(A)** and mortality **(B)** of COVID-19 patients during hospitalization.

The ROC curves for the individual cytokines as predictors for disease progression and mortality are shown in **Figures 5A, B**. IP-10 best predicted disease progression on days 0 to 3 of illness with an AUC of 0.81 (95% CI: 0.68 – 0.95), followed by IL-6 at 11 – 14 days of illness (AUC 0.67; 95% CI: 0.61 – 0.73) (**Figure 5A**; **Supplementary Table 4**). For predicting mortality, IP-10 had an AUC of 0.77 (95% CI: 0.70 – 0.84) at 11 to 14 days of illness. Beyond 14 days of illness, IL-6 predicted mortality with an AUC of 0.75 (95% CI: 0.68 – 0.82) while IL-18 had an AUC of 0.69 (95% CI: 0.60 – 0.77) at 11 to 14 days of illness (**Figure 5B**; **Supplementary Table 5**).

## 4 Discussion

The cohort in this study consists predominantly of patients with severe to critical COVID-19 disease (69%). This is higher compared to published data from the same institution (48.9%) and another tertiary referral center in Metro Manila (54.1%) even after excluding mild and asymptomatic patients (11, 35). The recorded mortality rate of 18% closely approximates the data from both reports, 15.1% in UP-PGH (11) and 20.8% in San Lazaro Hospital (35). The rate of disease progression was not reported in these local studies. But data from China show disease progression at 35.3% for patients with moderate and severe disease (36), similar to our study results at 35%. Given the high mortality and rates of progression, it is important to predict patients who will have poor outcomes to implement anticipatory care early in the course of the disease.

Older age, severe to critical illness on admission, existing comorbidities (DM, CKD), shortness of breath, and sensorial changes were associated with disease progression and mortality in this cohort. Age is an established risk factor for disease progression and mortality in multiple studies (10, 18, 33, 36, 37). Published data on comorbidities are variable but many studies reported DM and CKD to be associated with unfavorable outcomes among hospitalized patients with COVID-19 (1, 5, 18, 33, 36). Patients with severe disease were likely to die compared to their nonsevere counterparts. Expectedly, more nonsurvivors have received dexamethasone and tocilizumab. Local and international COVID-19 guidelines recommend the use of these agents to patients with severe disease (26, 38, 39). Corticosteroids have been shown to improve outcomes among patients with COVID-19 needing respiratory support (40), while tocilizumab on top of corticosteroids also improved outcomes also improved outcomes among patients with hypoxia and signs of systemic inflammation (41).

Our study showed that high D-dimer, high LDH, low ALC, and low albumin levels were associated with disease progression, while elevated D-dimer and LDH were associated with in-hospital mortality. These findings are consistent with other international and local reports on COVID-19 (11, 42–45). The finding of high D-dimer also provided evidence for SARS-CoV-2 infection as a hypercoagulable state (46), which further supports the use of prophylactic anticoagulation among patients with moderate to severe COVID-19. This hypercoagulable state can be attributed to neutrophil extracellular traps activation, which can trigger immunothrombosis in COVID-19 infection (47). In addition to what is already known, our study provided important insights about COVID-19: first, we provided the cut-off values and optimal time point for measurement of D-dimer (> 1 µg/mL), LDH, ALC (<1,100), and albumin (< 40 g/L); second, we reported cytokines that are significantly affected by COVID-19 and their use in predicting disease progression and mortality; third, we provided insights on the dynamics of IL-6 in the course of COVID-19 disease.

For routine laboratory tests, D-dimer >1 µg/mL and LDH >359.5 U/L obtained on the 4^th^ to 10^th^ day of illness may predict patients who are at high risk for disease progression and mortality, thus would warrant closer monitoring. Those who have ALC <1,100 and albumin <40 g/L should also be monitored due to high risk for mortality. Except for D-dimer, these are common tests available in most diagnostic facilities and hospitals. Incorporating them in clinical pathways for admitted patients with moderate to severe COVID-19 should not be a problem. As mentioned, high D-dimers are suggestive of a hypercoagulable state. It may be a marker of increased risk or even herald the presence of a thrombotic event. For D-dimer, our study does not only provide information on its predictive capacity, but also provides a research opportunity on the utility of D-dimer in guiding anticoagulant dosing among patients with COVID-19. A previous study showed that therapeutic-dose low molecular weight heparin decreased major thromboembolism and death compared with institutional standard heparin thromboprophylaxis among inpatients with COVID-19 with very elevated D-dimer levels (48). However, this benefit was only observed among noncritically ill patients with COVID-19 (48–50).

In addition to routine tests, our results agree with previous studies on the use of IP-10 as an early biomarker that distinguishes patients at risk for poor outcomes. Our results show clear divergence from day 0 to day 10 of illness – providing a wide window for clinicians as well as for researchers in terms of studying potential drug therapies. IP-10 is a protein secreted by various cell types such as monocytes, endothelial cells, and fibroblasts in response to IFNγ. IP-10 acts as a chemotactic factor for T cells, natural killer cells, monocytes, macrophages, and dendritic cells (51). IP-10 inhibits endothelial recovery independently of any other inflammatory factor, explaining the pervasive endothelialitis that is seen in severe and critical COVID-19 patients (52). Moreover, a critical factor for the exacerbation of the pathology of acute respiratory distress syndrome. It acts *via* autocrine signaling to promote oxidative burst and chemotaxis of inflamed neutrophils, leading to fulminant pulmonary inflammation (53). Neutrophilic infiltration has been observed post-mortem in pulmonary capillaries and alveolar space of patients who died from COVID-19 (54). Aside from the release of reactive oxygen species and cytokines to promote inflammation, neutrophils are known to produce neutrophil extracellular traps (NETs) composed of chromatin, anti-microbial proteins and oxidative enzymes meant to increase viscosity of respiratory tract mucus and eliminate pathogens (55). Although neutrophils act as first line of defense during infection of the lower respiratory tract, unregulated neutrophil activation can result to pneumonia and/or acute respiratory distress syndrome (56). Zuo et al. (57) has reported elevated NET markers such as myeloperoxidase-DNA complexes and citrullinated histone H3 (Cit-H3), the former of which was markedly elevated in hospitalized patients requiring mechanical ventilation (57).

A previous study also showed that IP-10 tends to elevate earlier in COVID-19 patients than other inflammatory cytokines (58). Several studies have shown that plasma levels of IP-10 are suitable biomarkers associated with the severity of COVID-19 diseases and may also be related to the risk of death in COVID-19 patients (59–62). Severe COVID-19 showed significantly higher levels of IP-10 than patients with mild COVID-19. Moreover, IP-10 levels were also positively correlated with SARS-CoV-2 titers in COVID-19 patients (63).

IL-6 is somewhat unique in that divergence was seen very early (0 – 3 days) and late (>14 days) in the course of the disease. This result highlights the usefulness of IL-6 as a prognostic biomarker for patients who presented to the hospital early or late in the course of the disease. This also shows that IL-6 can be used in monitoring patients with COVID-19 since IL-6 values can prognosticate disease progression and mortality even beyond 14 days after the onset of infection. However, IL-6 is only useful as a prognostic biomarker for patients not receiving tocilizumab. IL-6 is the main cytokine associated with several inflammatory diseases. Aside from being a pro-inflammatory cytokine, IL-6 promotes resistance to pathogens and tissue homeostasis (64). Previous studies showed that high serum levels of IL-6 are significantly related to adverse clinical outcomes such as admission to the intensive care unit, ARDS, and death (65, 66). Several studies recommended the early detection of serum IL-6 levels after admission to identify patients with the highest risk of disease progression and mortality (67–69).

IL-18, despite its “late” divergence, may still be useful especially for patients who present late in the course of their disease. IL-18 is a proinflammatory cytokine that facilitates IFN-γ production by Th1 cells. In the presence of IL-12, it also stimulates the production of IFN- by non-polarized T cells, NK cells, NKT cells, B cells, DC, and macrophages (70). A previous study in Brazil showed that fatal cases of COVID-19 had elevated levels of IL-18 (71). Multiple studies also showed that IL-18 is a marker of COVID-19 infection severity (71–73). Serum IL-18 levels in COVID-19 patients were noticeably higher compared to healthy controls, peaking in the group with severe pneumonia (74). IL-18 participates as an element of inflammasome activation among COVID-19 patients. Inflammasomes play a key role in the innate immune response to pathogen-associated molecular patterns. Inflammasomes release proinflammatory cytokines like IL-1 and IL-18 to the extracellular environment causing inflammation and cell death. Overactivation of this process leads to exacerbation of COVID-19 infection (75, 76).

Our study has several limitations given its observational nature. First, the choice, the timing, and the frequency of routine laboratory tests were at the discretion of the attending physicians. Thus, it is likely that patients with more severe disease underwent extensive laboratory evaluation, earlier testing, and more frequent testing than patients with nonsevere disease. Second, the majority of the patients were admitted beyond four days of illness, hence fewer laboratory data were available for days 0 to 3. Although this could have missed significant deviation early in the course of the disease, our data reflected the most clinically relevant time point - *the time when most patients seek medical attention*. Third, we used cut-off values based on published studies for routine laboratory tests. Fourth, the COVID-19 pandemic is evolving along with the availability of vaccines and therapeutic agents. The vaccine roll-out began in March 2021. We were able to include therapeutic agents as shown in **Table 1**, but this is limited to those available and used in the hospital. Some patients may have tried “other agents” such as ivermectin, herbal supplements, and high dose vitamins – and the effect of such therapies cannot be controlled or considered in the analysis. Unfortunately, we were also unable to obtain the COVID-19 vaccination history of the patients. Cardiac magnetic resonance imaging was not done in any of the patients, which makes it very difficult to distinguish between acute myocardial infarction and myocarditis. Our study also had missing data. Some laboratory markers were not obtained from patients, or the values of the cytokines were beyond the range of detection. We did not run multiple imputations for these missing data. And finally, our study was conducted during the earlier waves of SARS-CoV-2, with the more virulent variants as opposed to the seemingly milder Omicron variant (77).

Future studies could explore the cost-effectiveness of these tests, especially since cytokine level determination is not widely available locally. For the biomarkers, monitoring trends before, during, and after treatment may be valuable in better understanding the immunopathologic mechanisms of COVID-19, as well as their association with clinical outcomes. These biomarkers may be studied as a predictor of the need for a higher FiO2 requirement. This can help clinicians better respond to acute complications, long-term sequelae, and possible future COVID-19 variants. This study emphasizes the need to develop research centers that can explore these critical immunologic mechanisms and be agile to respond to future pandemics and similar public health threats.

## 5 Conclusions

Among patients with moderate to severe COVID-19, elevated D-dimer (>1 µg/mL) or LDH (>359.5 U/L) obtained within ten days of illness onset were associated with disease progression and mortality. Additionally, lymphopenia and hypoalbuminemia were also associated with disease progression. For cytokines, elevated IP-10 within 14 days of illness, and elevated IL-6 beyond 14 days of illness were associated with disease progression and mortality. High levels of IL-18 on the 11^th^ to 14^th^ day of illness were also associated with mortality. Timely utilization of these biomarkers will better guide patient monitoring and management.

## Data availability statement

The original contributions presented in the study are included in the article/**Supplementary Material**. Further inquiries can be directed to the corresponding author.

## Ethics statement

This study was approved by the UP Manila Research Ethics Board (UPMREB 2020-251-01). The patients/participants provided their written informed consent to participate in this study.

## Author contributions

Conceptualization: FP, JA, SP-S, AMo, AMa, JT, OT, EQ, MU, RL, JR, AD-W, and MA. Methodology: FP, JA, SP-S, AMo, AMa, JT, OT, EQ, MU, RL, JR, MM, KA, JP, AD-W, and MA. Formal analysis: FP, JA, SP-S, AMo, AMa, JT, OT, EQ, MU, RL, JR, MM, KA, AA, AD-W, and MA. Investigation: FP, JA, SP-S, AMo, AMa, JT, OT, EQ, MU, RL, JR, MM, KA, AD-W, and MA. Resources: FP, SP-S, and MA. Data curation: JA, SP-S, JT, OT, EQ, MU, RL, JR, MM, KA, and JP. Writing – original draft preparation: FP, JA, SP-S, AMo, AMa, JT, OT, EQ, MU, RL, JR, and MM. Writing – review & editing: FP, JA, SP-S, AMo, AMa, OT, MM, and MA. Supervision: FP, JA, SP-S, AMo, AMa, AD-W, and MA. Project administration: FP, JA, SP-S, AD-W, and MA. Funding acquisition: FP, JA, SP-S, AMo, AMa, JT, OT, EQ, MU, RL, JR, AD-W, and MA. All authors contributed to the article and approved the submitted version.
