# Supplementary material for: Utility of laboratory and immune biomarkers in predicting disease progression and mortality among patients with moderate to severe COVID-19 disease at a Philippine tertiary hospital
Source: Front Immunol. 2023 Feb 28;14:1123497. doi: 10.3389/fimmu.2023.1123497 (PMC10011458; doi:10.3389/fimmu.2023.1123497)
Supplement: Supplementary file 1 [file DataSheet_1.docx]

**SUPPLEMENTARY TABLES**

**Supplementary Table 1.** Summary of Laboratory Predictors of Adverse Outcomes.

| **TEST** | **OUTCOME** | **ASSOCIATION** | **Ref** |
| --- | --- | --- | --- |
| **Hematologic** | | | |
| Neutrophils | Mortality | high neutrophils: HR 1.08 (1.01-1.17; p = 0.03) | (Wu *et al*., 2020) |
|  | ARDS | high neutrophils: HR 1.14 (1.09-1.19; p < 0.001) | (Wu *et al*., 2020) |
| Neutrophil  Lymphocyte Ratio  (NLR) | Severe Illness | NLR>3 has worse prognosis  (p = 0.0005) | (J. Liu *et al.*, 2020a) |
| Platelet | Mortality | Thrombocytopenia: OR 5.1 (1.8-14.6) | (Lippi *et al*., 2020) |
| Platelet Lymphocyte Ratio (PLR) | Severe Illness | Higher PLR at peak platelet during treatment was an independent  influencing factor in severe patients (p<0.05) | (L. Chen *et al*., 2020) |
| **Biochemical & Infection-related Indices** | | | |
| Albumin | Disease  progression | Albumin < 40 g/L: OR 7.35 (1.098- 50.0, p=0.003) | (W. Liu *et al*., 2020) |
| Total bilirubin (TB) | Mortality | high TB: HR 1.07 (1.02-1.12; p = 0.003) | (Wu *et al*., 2020) |
|  | ARDS | high TB: HR 1.05 (1.02-1.08; p = 0.001) | (Wu *et al*., 2020) |
| AST | ARDS | high AST: HR 1.02 (1.01-1.03; p 0 < 0.001 | (Wu *et al*., 2020) |
| Procalcitonin | Mortality | High Procalcitonin: OR 13.75 (1.81- 104.4, p=0.011) | (F. Zhou *et al*., 2020) |
| Hs-Trop-I | Mortality | Trop >28: OR 80.07 (10.34-620.36, p=0.001) | (F. Zhou *et al*., 2020) |
| LDH | Mortality | LDH > 100 U/L: HR 1.30 (1.11-1.52; p = 0.001) | (Wu *et al.*, 2020) |
|  | ARDS | LDH > 100 U/L: HR 1.61 (1.44-1.79; p < 0.001) | (Philippine Society for  Microbiology and  Infectious Diseases,  2020b; Wu *et al*., 2020) |
| Glucose | ARDS | high glucose: HR 1.13 (1.08-1.19; p < 0.001) | (Wu *et al*., 2020) |
| Creatinine | ARDS | creatine > 10uM: HR 1.05 (1.01-1.10; p = 0.02) | (Wu *et al*., 2020) |
| hs CRP | ARDS | hs CRP >5mg/ml: HR 4.81 (1.52-15.27; p = 0.008) | (Wu *et al*., 2020) |
| CRP | Mortality | CRP 125 mg/L in deaths versus 40 mg/L in survivors | (F. Zhou *et al*., 2020) |
|  | Disease  Progression | CRP >8.2 mg/dL: OR 10.53 (1.24-34.7, p=0.028) | (W. Liu *et al*., 2020) |
| Ferritin | Mortality | Ferritin >300 ug/L: OR 9.10 (2.04- 40.58, p=0.0038); HR 3.53 (1.52-8.16; p = 0.003) | (F. Zhou *et al*., 2020) |
| D-Dimer | Mortality | D-Dimer > 1 ug/mL: OR 18.42 (2.64- 128.55, p=0.003) | (F. Zhou *et al*., 2020) |
|  | Mortality | High D-Dimer: 1.02 (1.01-1.04; p = .002) | (Wu *et al*., 2020) |
|  | ARDS | High D-Dimer: 1.03 (1.01-1.04; p <.001 | (Wu *et al*., 2020) |

**References**

Lippi G, Plebani M, Michael Henry B. Thrombocytopenia is associated with severe coronavirus disease 2019 (COVID-19) infections: A meta-analysis [published online ahead of print, 2020 Mar 13]. *Clin Chim Acta.*2020;S0009-8981(20)30124-8. doi:10.1016/j.cca.2020.03.022

Liu J, Li S, Liu J, Liang B, Wang X, Wang H, et al. Longitudinal characteristics of lymphocyte responses and cytokine profiles in the peripheral blood of SARS-CoV-2 infected patients. EBioMedicine [Internet]. 2020 May 1 [cited 2022 Nov 8];55. Available from: <https://pubmed.ncbi.nlm.nih.gov/32361250/>

Philippine Society for Microbiology and Infectious Diseases. (2020b). *Should laboratory markers be used for early prediction of severe and possibly fatal COVID-19?* PSMID. https://www.psmid.org/should-laboratory-markers-be used-for-early-prediction-of-severe-and-possibly-fatal-covid-19-2/

Wu C, Chen X, Cai Y, Xia J, Zhou X, Xu S, et al. Risk Factors Associated With Acute Respiratory Distress Syndrome and Death in Patients With Coronavirus Disease 2019 Pneumonia in Wuhan, China. JAMA Intern Med [Internet]. 2020 Jul 1 [cited 2022 Nov 8];180(7):934–43. Available from: https://pubmed.ncbi.nlm.nih.gov/32167524/

Zhou, F., Yu, T., Du, R., Fan, G., Liu, Y., Liu, Z., Xiang, J., Wang, Y., Song, B., Gu, X., Guan, L., Wei, Y., Li, H., Wu, X., Xu, J., Tu, S., Zhang, Y., Chen, H., & Cao, B. (2020). Clinical course and risk factors for mortality of adult inpatients with COVID-19 in Wuhan, China: A retrospective cohort study. *The Lancet*, S0140673620305663. https://doi.org/10.1016/S0140-6736(20)30566-3

**Supplement Table 2.** Adjusted Estimates for Cytokine Levels and Disease Progression

| **Cytokine (N)** | **Ill day** | **Without Disease Progression**  Median, ng/mL and Interquartile Range | **With Disease Progression**  Median, ng/mL and Interquartile Range | **p-value** |
| --- | --- | --- | --- | --- |
| IFN-y (266) | 0 – 3 | 74.17 (-62.13 – 210.47) | 62.85 (-82.19 – 207.89) | 0.39 |
|  | 4 – 10 | 82.67 (42.32 – 123.01) | 35.16 (-19.53 – 89.84) |  |
|  | 11 – 14 | 83.18 (39.01 – 127.35) | 109.34 (47.26 – 171.41) |  |
|  | >14 | 102.92 (64.27 – 141.57) | 87.27 (25.46 – 149.08) |  |
|  | **p-value** | 0.70 | |  |
| TNF-a (290) | 0 – 3 | 345.13 (-2.15 – 692.41) | 213.53 (-187.01 – 614.06) | 0.18 |
|  | 4 – 10 | 194.37 (79.24 – 309.49) | 188.95 (32.27 – 345.62) |  |
|  | 11 – 14 | 212.10 (91.18 – 333.01) | 237.78 (66.61 – 408.95) |  |
|  | >14 | 324.34 (216.76 – 431.91) | 364.00 (189.52 – 538.48) |  |
|  | **p-value** | 0.83 | |  |
| IL – 8 (307) | 0 – 3 | 692.53 (-462.18 – 1847.25) | 560.02 (-761.18 – 1881.22) | 0.43 |
|  | 4 – 10 | 897.58 (492.65 – 1302.51) | 587.06 (20.99 – 1153.12) |  |
|  | 11 – 14 | 1259.39 (843.48 - 1675.30) | 864.16 (261.12 – 1467.21) |  |
|  | >14 | 1159.20 (777.78 – 1540.62) | 787.70 (163.79 – 1411.61) |  |
|  | **p-value** | 0.36 | |  |
| IL-6  (307) | 0 – 3 | 441.50 (-450.32 – 1333.31) | 1402.29 (389.67 – 2414.91) | 0.06 |
|  | 4 – 10 | 315.07 (24.62 – 605.53) | 397.65 (-5.55 – 800.86) |  |
|  | 11 – 14 | 489.44 (188.66 – 790.22) | 534.03 (97.92 – 970.14) |  |
|  | >14 | 359.34 (89.03 – 629.64) | 1248.50 (798.47 – 1698.52) |  |
|  | **p-value** | 0.03 | |  |
| IL-1B (205) | 0 – 3 | 122.05 (-28.72 – 272.81) | 52.73 (-117.25 – 222.70) | 0.70 |
|  | 4 – 10 | 67.89 (13.60 – 122.17) | 136.44 (47.69 – 225.18) |  |
|  | 11 – 14 | 129.86 (63.35 – 196.37) | 150.08 (46.83 – 253.33) |  |
|  | >14 | 136.61 (86.04 – 187.19) | 123.76 (41.56 – 205.96) |  |
|  | **p-value** | 0.97 | |  |
| IL – 2  (193) | 0 – 3 | 17.35 (-3.49 – 38.19) | 11.71 (-12.26 – 35.67) | 0.95 |
|  | 4 – 10 | 8.81 (1.66 – 15.95) | 12.03 (0.74 – 23.32) |  |
|  | 11 – 14 | 12.62 (3.85 – 21.38) | 10.04 (-2.69 – 22.78) |  |
|  | >14 | 14.23 (6.63 – 21.83) | 11.36 (0.23 -22.48) |  |
|  | **p-value** | 0.71 | |  |
| IL-4  (66) | 0 – 3 | 147.99 (5.61 – 290.37) | 158.88 (-53.08 – 370.85) | 0.59 |
|  | 4 – 10 | 20.26 (-17.94 – 58.46) | 98.11 (9.62 – 186.59) |  |
|  | 11 – 14 | 69.93 (14.30 – 125.57) | 59.82 (-28.20 – 147.85) |  |
|  | >14 | 57.22 (-0.33 – 114.78) | 67.36 (-10.23 – 144.95) |  |
|  | **p-value** | 0.60 | |  |
| **IP-10**  (305) | 0 – 3 | 9872.65 (-1707.03 – 21452.33) | 43532.10 (30336.84 – 56727.37) | **<0.00001** |
|  | 4 – 10 | 10560.48 (6633.92 – 14487.03) | 21077.02 (15567.53 – 26586.52) |  |
|  | 11 – 14 | 6282.21 (2204.15 – 10360.26) | 18827.85 (12964.94 – 24690.76) |  |
|  | >14 | 3021.96 (-703.33 – 6747.25) | 2661.90 (-3399.41 – 8723.22) |  |
|  | **p-value** | <0.00001 | |  |
| IL – 18  (291) | 0 – 3 | 200.10 (-87.43 – 487.64) | 206.05 (-119.38 – 531.49) | 0.21 |
|  | 4 – 10 | 190.77 (92.72 – 288.83) | 192.77 (56.98 – 328.55) |  |
|  | 11 – 14 | 180.33 (72.45 – 288.20) | 464.28 (315.55 – 613.02) |  |
|  | >14 | 212.67 (121.81 – 303.54) | 286.37 (139.55 – 433.20) |  |
|  | **p-value** | 0.20 | |  |
| IL-10 (151) | 0 – 3 | 303.64 (18.39 – 588.89) | 88.42 (-147.75 – 324.59) | 0.92 |
|  | 4 – 10 | 162.01 (65.53 – 258.48) | 130.28 (-1.83 -262.40) |  |
|  | 11 – 14 | 119.18 (2.63 – 235.73) | 132.49 (-20.85 – 285.83) |  |
|  | >14 | 114.44 (17.66 – 211.23) | 183.53 (30.91 – 336.15) |  |
|  | **p-value** | 0.56 | |  |

**Supplement Table 3.** Adjusted Estimates for Cytokine Levels and Mortality

| **Cytokine (N)** | **Ill day** | **Survivor**  Median, ng/mL and Interquartile Range | **Non-Survivor**  Median, ng/mL and Interquartile Range | **p-value** |
| --- | --- | --- | --- | --- |
| IFN-y (266) | 0 – 3 | 61.29 (-44.59 – 167.17) | 117.51 (-153.00 – 388.02) | 0.27 |
|  | 4 – 10 | 73.70 (38.37 – 109.03) | 27.64 (-51.09 – 106.37) |  |
|  | 11 – 14 | 79.94 (40.50 – 119.37) | 145.31 (60.96 – 229.66) |  |
|  | >14 | 103.76 (-153.00 – 137.97) | 48.61 (-59.68 – 156.90) |  |
|  | **p-value** | 0.91 | |  |
| IL – 8 (307) | 0 – 3 | 680.61(-270.27 – 1631.48) | 512.23 (--1580.40 – 2604.86) | 0.91 |
|  | 4 – 10 | 868.05 (512.22 – 1223.89) | 414.45 (-414.59 – 1243.49) |  |
|  | 11 – 14 | 1256.71 (889.48 – 1623.95) | 404.07 (-489.90 – 1298.03) |  |
|  | >14 | 1130.22 (788.87 – 1471.56) | 465.00 (-558.68 – 1488.68) |  |
|  | **p-value** | 0.21 | |  |
| **IL-6**  (307) | 0 – 3 | 523.47 (-183.69 – 1230.63) | 2454.46 (906.30 – 4002.63) | **<0.00001** |
|  | 4 – 10 | 321.44 (74.50 – 568.39) | 421.17 (-146.30 – 988.63) |  |
|  | 11 – 14 | 414.38 (157.50 – 671.26) | 1014.34 (389.60 – 1639.08) |  |
|  | >14 | 329.69 (94.97 – 564.40) | 3037.35 (2320.95 – 3753.76) |  |
|  | **p-value** | <0.00001 | |  |
| IL-1B  (205) | 0 – 3 | 90.78 (-33.05 – 214.61) | 85.69 (-171.88 – 343.27) | 0.91 |
|  | 4 – 10 | 88.38 (38.36 – 138.41) | 78.27 (-45.27 – 201.80) |  |
|  | 11 – 14 | 144.11 (84.13 – 204.10) | 91.07 (-59.31 – 241.45) |  |
|  | >14 | 137.68 (92.66 – 182.69) | 77.17 (-71.18 – 225.52) |  |
|  | **p-value** | 0.54 | |  |
| IL – 2  (193) | 0 – 3 | 14.72 (-3.87 – 33.31) | 15.35 (-14.26 – 44.95) | 0.96 |
|  | 4 – 10 | 9.60 (3.16 – 16.04) | 10.69 (-6.32 – 27.70) |  |
|  | 11 – 14 | 12.29 (4.17 – 20.40) | 9.97 (-5.41 – 25.36) |  |
|  | >14 | 13.68 (6.96 – 20.41) | 10.86 (-6.61 – 28.34) |  |
|  | **p-value** | 0.89 | |  |
| IL-4  (66) | 0 – 3 | 139.28 (-0.94 – 279.50) | 163.18 (-55.05 – 381.41) | 0.64 |
|  | 4 – 10 | 31.73 (-4.78 – 68.24) | 80.57 (-61.04 – 222.18) |  |
|  | 11 – 14 | 63.54 (10.05 – 117.03) | 67.37 (-30.88 -165.62) |  |
|  | >14 | 59.33 (12.25 – 106.41) | 84.79 (-59.82 – 229.41) |  |
|  | **p-value** | 0.60 | |  |
| **IL – 18**  (291) | 0 – 3 | 192.59 (-37.41 – 422.59) | 235.05 (-268.43 – 738.53) | **<0.001** |
|  | 4 – 10 | 180.92 (97.07 – 264.77) | 244.08 (46.92 – 441.25) |  |
|  | 11 – 14 | 172.84 (81.39 – 264.30) | 890.36 (668.33 – 1112.40) |  |
|  | >14 | 216.73 (137.47 – 296.00) | 385.63 (147.34 – 623.92) |  |
|  | **p-value** | <0.01 | |  |
| **IP-10**  (305) | 0 – 3 | 17235.82 (7903.67 – 26567.97) | 57367.50 (36903.53 – 77831.46) | **<0.00001** |
|  | 4 – 10 | 12193.98 (8803.96 – 15583.99) | 24424.94 (16597.45 – 32252.43) |  |
|  | 11 – 14 | 5656.98 (2133.65 – 9180.31) | 36941.07 (28421.85 – 45460.29) |  |
|  | >14 | 2605.78 (-658.35 – 5869.92) | 4624.30 (-5141.66 – 14390.26) |  |
|  | **p-value** | <0.00001 | |  |
| IL-10  (151) | 0 – 3 | 183.64 (-27.02 – 394.30) | 152.32 (-219.65 – 524.31) | 0.99 |
|  | 4 – 10 | 159.78 (72.85 – 246.71) | 112.74 (-61.98 – 287.47) |  |
|  | 11 – 14 | 112.60 (9.23 – 215.98) | 168.62 (-40.45 – 377.69) |  |
|  | >14 | 152.33 (44.61 – 223.28) | 137.39 (-61.31 – 336.10) |  |
|  | **p-value** | 0.96 | |  |

**Supplementary Table 4.** AUC calculation for IL-6 and IP-10 in disease progression group of COVID-19 patients.

| **Days of Illness** | **IL-6** | | **IP-10** | |
| --- | --- | --- | --- | --- |
|  | **AUC** | **95%CI** | **AUC** | **95%CI** |
| 0-3 days | 0.56 | 0.38 - 0.74 | 0.81 | 0.68 - 0.94 |
| 4-10 days | 0.63 | 0.58 - 0.68 | 0.56 | 0.51 - 0.62 |
| 11-14 days | 0.67 | 0.61 - 0.73 | 0.63 | 0.57 - 0.69 |
| >14 days | 0.6 | 0.55 - 0.66 | 0.58 | 0.53 - 0.64 |

**Supplementary Table 5.** AUC calculation for IL-6, IL-18, and IP-10 in nonsurvivors group of COVID-19 patients.

| **Days of Illness** | **IL-6** | | **IL-18** | | **IP-10** | |
| --- | --- | --- | --- | --- | --- | --- |
|  | **AUC** | **95%CI** | **AUC** | **95%CI** | **AUC** | **95%CI** |
| 0-3 days | 0.58 | 0.33 - 0.83 | 0.52 | 0.27 - 0.76 | 0.65 | 0.40 - 0.91 |
| 4-10 days | 0.65 | 0.59 - 0.71 | 0.66 | 0.60 - 0.73 | 0.54 | 0.46 - 0.62 |
| 11-14 days | 0.75 | 0.68 - 0.82 | 0.69 | 0.60 - 0.77 | 0.77 | 0.70 to 0.84 |
| >14 days | 0.75 | 0.68 - 0.82 | 0.62 | 0.52 - 0.72 | 0.66 | 0.59 - 0.74 |

**SUPPLEMENTARY FIGURES**

**
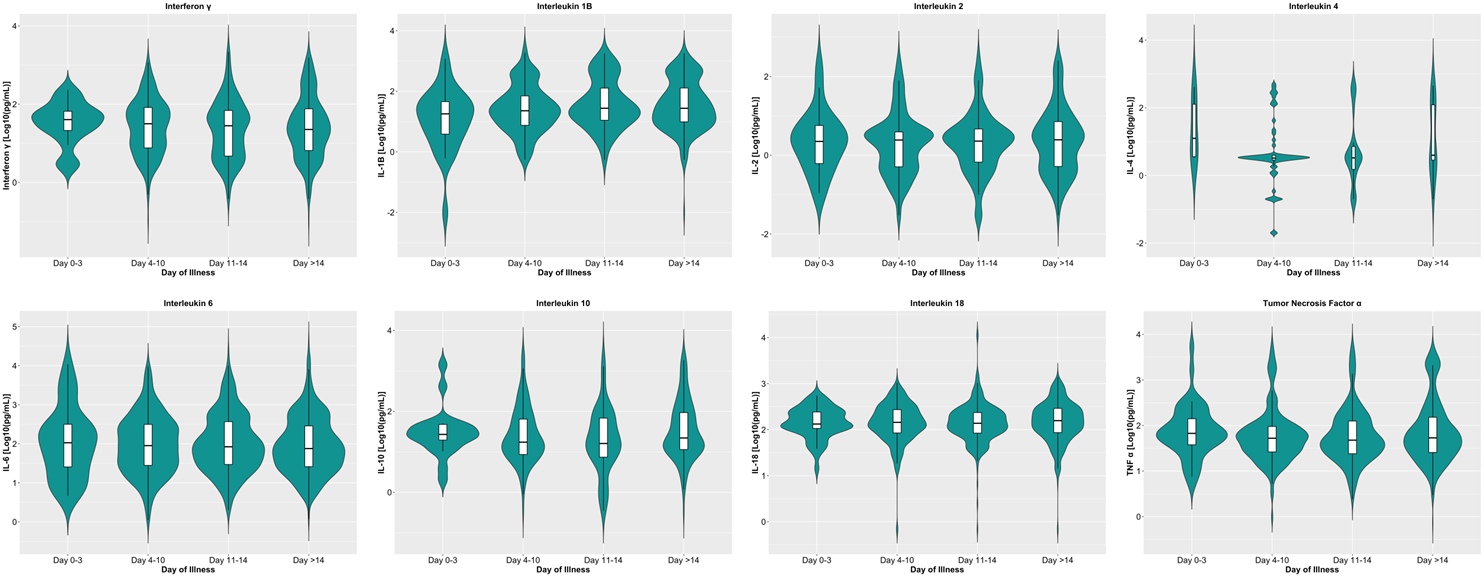
**

**Supplementary Figure 1.** Dynamics of serum cytokine levels during the disease course in the general COVID-19 patient cohort. The cytokines were measured in terms of days from illness onset. All samples collected from 400 patients were stratified into four intervals starting from illness onset. The dots represent individual measurement, and the box plots represent medians with interquartile range. The different groups were compared using the Kruskal-Wallis test with Dunn's post hoc test.


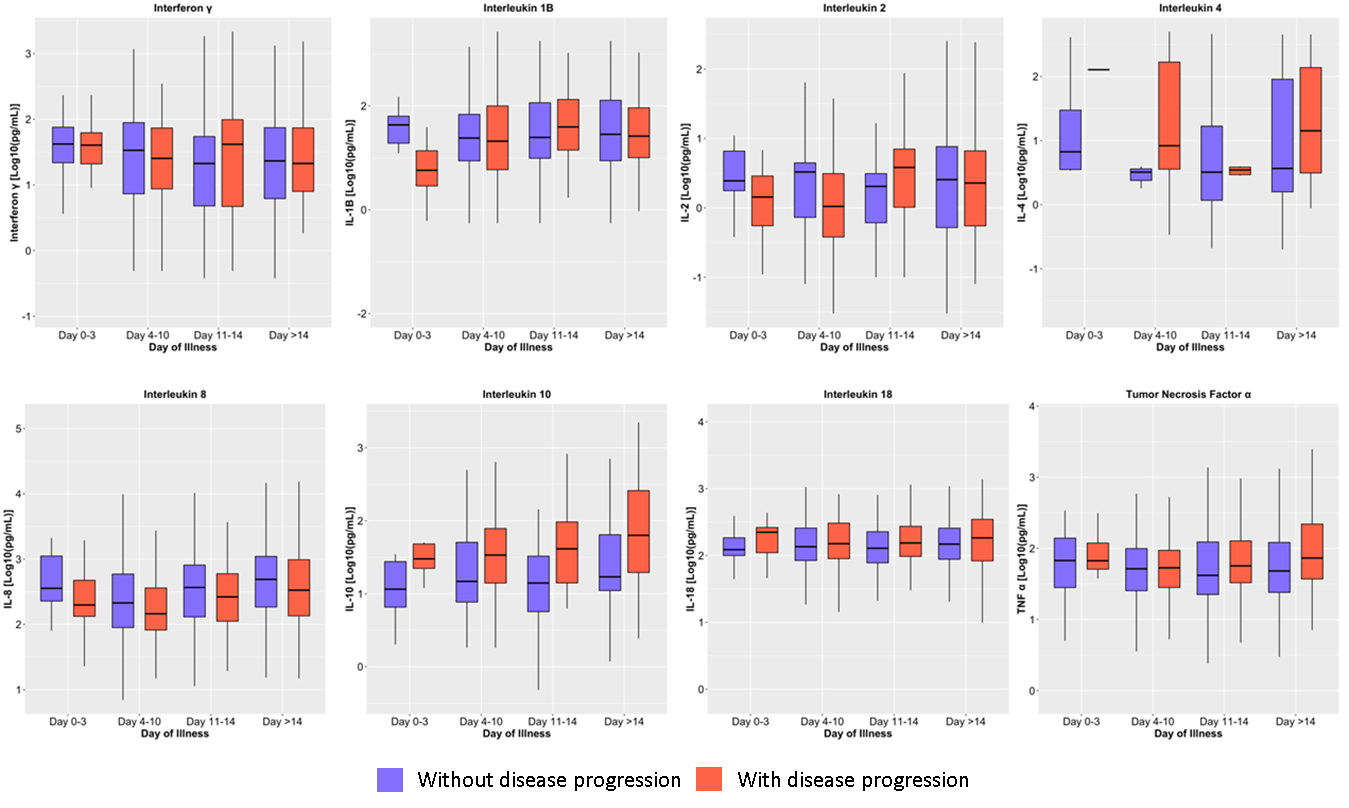


**Supplementary Figure 2.** Dynamics of serum cytokine levels during the disease course in COVID-19 patients based on disease progression. The cytokines were measured in terms of days from illness onset. All samples collected from 400 patients were stratified into four intervals starting from illness onset. The dots represent individual measurements, and the box plots represent medians with interquartile range. The different groups were compared by repeated measures mixed model regression with post hoc test. *p<0.05, **p<0.01, ***p<0.001, ****p<0.0001.


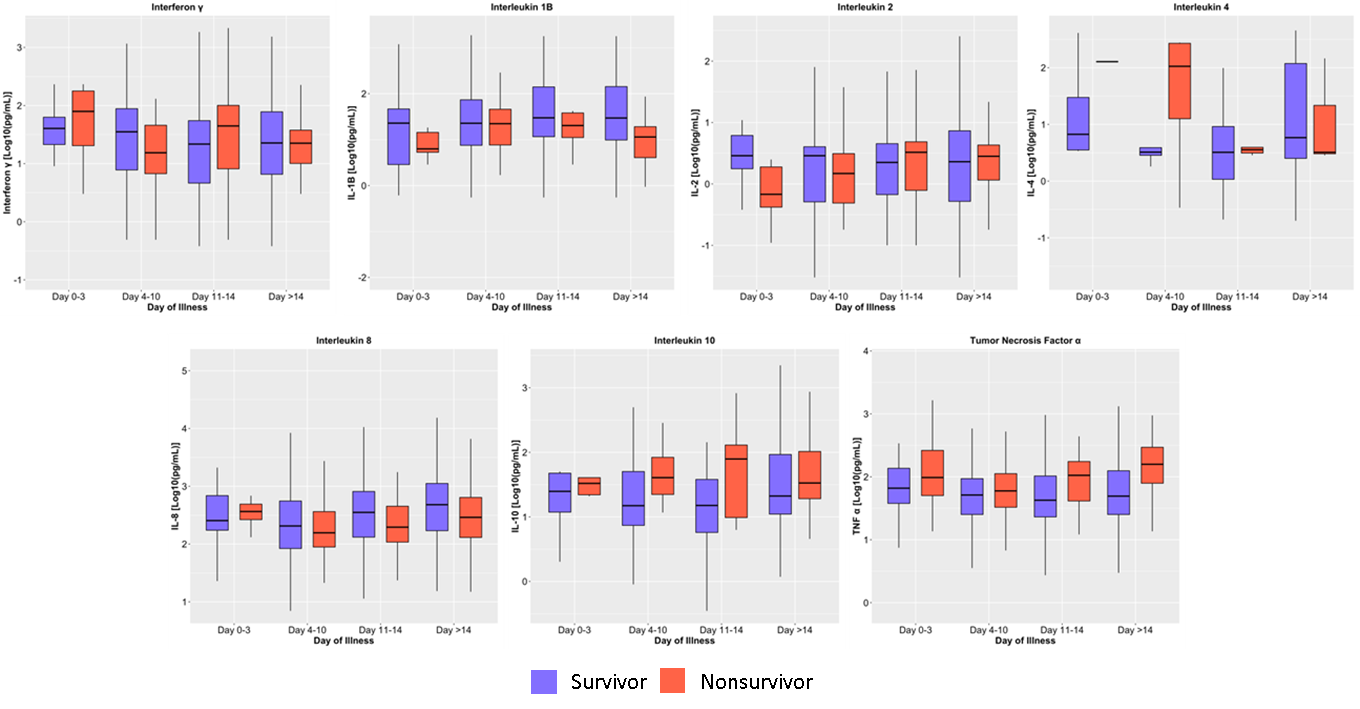


**Supplementary Figure 3.** Dynamics of serum cytokine levels during the disease course in COVID-19 patients based on mortality. The cytokines were measured in terms of days from illness onset. All samples collected from 400 patients were stratified into four intervals starting from illness onset. The dots represent individual measurements, and the box plots represent medians with interquartile range. The different groups were compared by repeated measures mixed model regression with post hoc test. *p<0.05, **p<0.01, ***p<0.001, ****p<0.0001


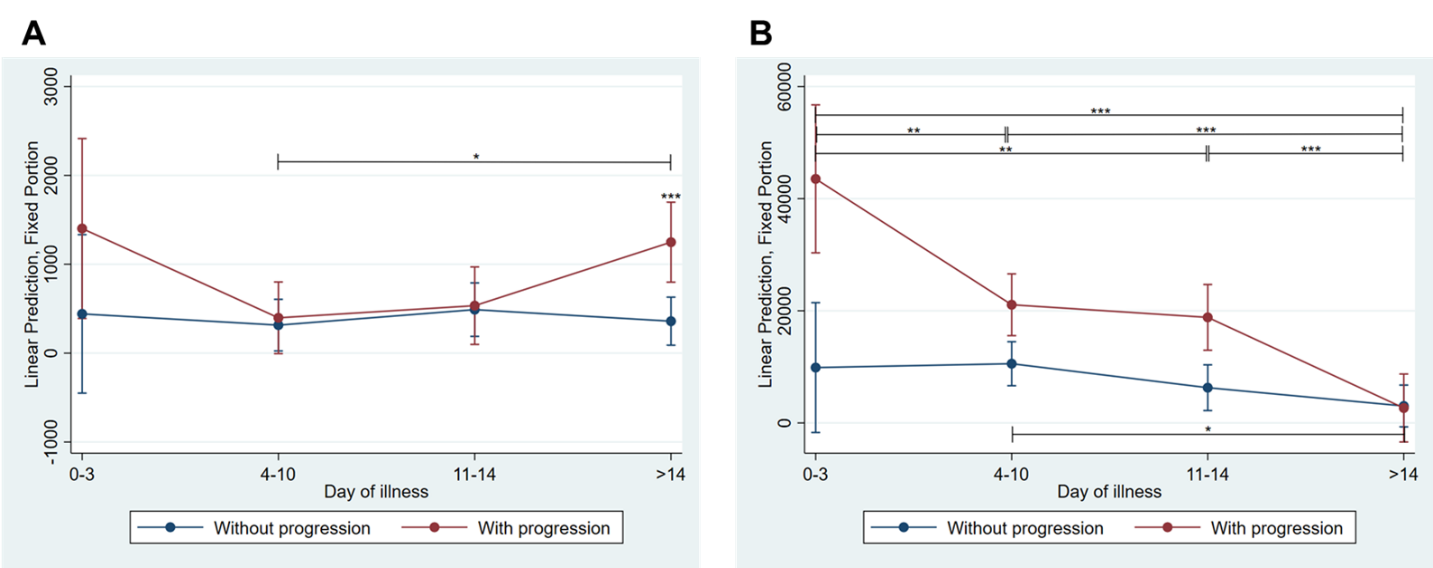


**Supplementary Figure 4.** Average prediction of (A) IL-6 and (B) IP-10 values with 95% CI between those with and without disease progression. The values were adjusted for the treatment received (i.e., tocilizumab or dexamethasone), presence of comorbidities (i.e., chronic kidney disease and diabetes mellitus), and age (cut off at 55 years old). * *p* < 0.05; ** *p* < 0.01; *** *p* < 0.001.


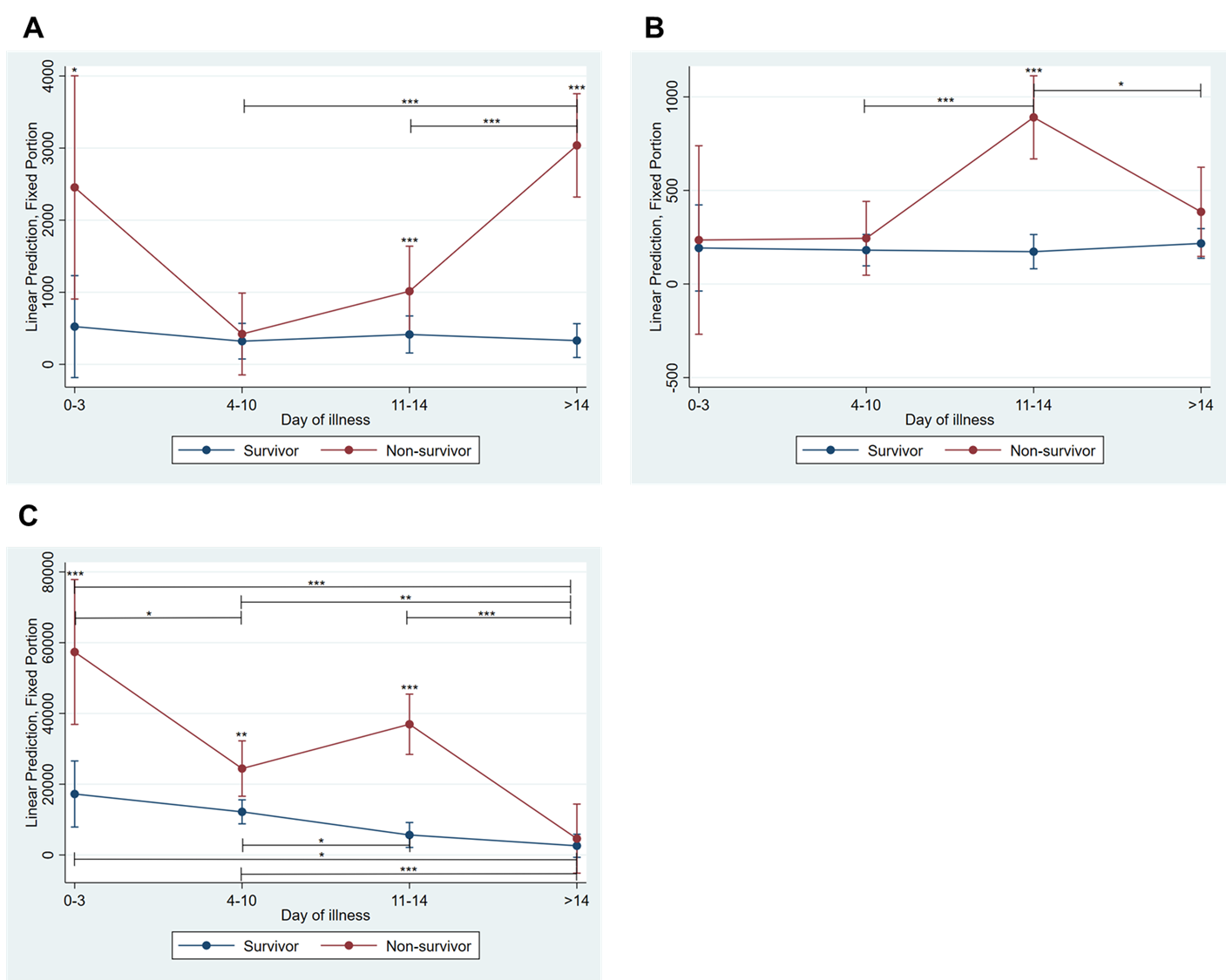


**Supplementary Figure 5.** Average prediction of (A) IL-6, (B) IL-18, and (C) IP-10 values with 95% CI between survivors and non-survivors. The values were adjusted for the treatment received (i.e., tocilizumab or dexamethasone), presence of comorbidities (i.e., chronic kidney disease and diabetes mellitus), and age (cut off at 55 years old). * *p* < 0.05; ** *p* < 0.01; *** *p* < 0.001.
